# COVID-19 vaccination and use of antibiotics in COVID-19 patients: a systematic review and meta-analysis

**DOI:** 10.1016/j.infpip.2025.100461

**Published:** 2025-06-03

**Authors:** Marios Politis, Ioanna Chatzichristodoulou, Varvara A. Mouchtouri, Georgios Rachiotis

**Affiliations:** a Department of Global Public Health, Karolinska Institute, Stockholm, Sweden; b Center for Epidemiology and Community Medicine, Stockholm Health Care Services, Stockholm, Sweden; c Department of Hygiene and Epidemiology, School of Medicine, University of Thessaly, Larisa, Greece; d Department of Internal Medicine, Norfolk and Norwich University Hospital, Norwich, United Kingdom

**Keywords:** COVID-19 vaccine, Antibiotics, Antimicrobial resistance

## Abstract

**Background:**

Vaccinations are considered one of the most effective medical interventions. Among other benefits, certain vaccinations help reduce antimicrobial resistance by decreasing antibiotic use. Considering reports of increased antimicrobial resistance during the COVID-19 pandemic, this study aimed to explore the relationship between COVID-19 vaccination status and antibiotic use in COVID-19 patients.

**Methods:**

A systematic literature search was conducted in PubMed, Scopus, Web of Science, Embase, and Google Scholar between January 1, 2021, and November 6, 2024. The included studies were assessed for risk of bias using the Newcastle-Ottawa tool. Narrative synthesis and random-effects meta-analysis were employed to synthesize the evidence.

**Results:**

Eight studies were included in this systematic review and meta-analysis (134,022 participants). COVID-19 vaccination was significantly associated with a 34% reduction in the odds of antibiotic use (OR: 0.662; 95% CI: 0.540–0.811) in COVID-19 patients. These findings were supported by the sensitivity analyses. In the subgroup analysis, a significant negative association was observed between COVID-19 vaccination and antibiotic use among COVID-19 patients across all study designs. A major limitation of this study is that most of the included studies did not adjust for confounders.

**Conclusions:**

COVID-19 vaccination was associated with a significant reduction in antibiotic use among COVID-19 patients. COVID-19 vaccination status may have influenced healthcare providers' decisions regarding antibiotic use in this group. Further large-scale cohort studies are needed to confirm these findings.

**Other:**

The study protocol is registered with PROSPERO (ID: CRD42023449625). No funding was provided for this study. The APCs were covered by the Karolinska Institute.

## Introduction

Vaccination is considered among the most cost-efficient and effective medical interventions preventing individuals from disease, disability, and death [1,2]. Today, 3.5–5 million deaths are prevented every year due to the global use of vaccinations against more than twenty life-threating diseases [3]. Vaccination also protects unvaccinated and vulnerable individuals by preventing infection spread to the community - a phenomenon known as population or herd immunity [1,4]. Beyond health benefits, vaccination results in enormous developmental, fiscal, and societal advantages [1,5,6]. Investment in vaccination programs has proven to be highly effective in high-, middle-, and low-income countries, providing significant development dividends and reducing inequalities both between and within countries [1,5].

Antimicrobial Resistance (AMR), the result of bacteria, parasites, viruses, and fungi being exposed to antimicrobial drugs, has been recognized by the World Health Organization (WHO) as one of the top ten global public health concerns playing a crucial negative role on the prevention and treatment of a wide range of infectious diseases [7,8]. Vaccinations decrease the use of antibiotics and subsequent AMR by preventing disease infections and their associated complications as well as by reducing the irrational use of antibiotics [5,[9], [10], [11], [12], [13], [14], [15], [16]]. While all vaccines are designed to prevent specific diseases, their benefits extend far beyond this, as certain vaccines also provide protection against diseases other than those they were initially intended to address [5]. For instance, it is well established that influenza vaccination offers indirect protection from bacterial infections in influenza patients, such as bacterial pneumonia and otitis media [5].

Vaccination status with certain vaccines has been found to be an important factor affecting antibiotic use and/or prescriptions. A meta-analysis of randomized controlled trials (RCTs) showed that influenza vaccination reduced antimicrobial prescriptions or days of antibiotic use with a ratio of means (RoM) of 0.71 (95% CI: 0.62–0.83), while also decreasing the proportion of people receiving antibiotics with a risk ratio (RR) of 0.63 (95% CI: 0.51–0.79) [16]. According to the same study, in a meta-analysis of RCTs, pneumococcal vaccination decreased the number or days of antibiotic prescriptions in children, with a RoM of 0.94 (95% CI: 0.92–0.96) [16]. The same trend was reported for both pneumococcal and influenza vaccines based on the results of 69 observational studies [16]. However, the authors noted that they did not identify any relevant studies on the relationship between COVID-19 vaccination and antibiotic use as of their search on December 1, 2021 [16]. Still, emerging evidence indicates that COVID-19 vaccination is also associated with reduced antibiotic prescriptions in older adults [17,18].

Despite the viral nature of COVID-19 and the relatively low prevalence of bacterial co-infections and secondary bacterial infections in COVID-19 patients (5.3%; 95% CI: 3.8–7.4% and 18.4%; 95% CI: 14.0–23.7%, respectively), antibiotics were prescribed in 74.6% (95% CI: 68.3–80.0%) of COVID-19 patients and were the most frequently reported drug used for COVID-19-related self-medication [[19], [20], [21]]. According to reports from the Centers for Disease Control and Prevention (CDC) and the Pan American Health Organization (PAHO), as well as evidence from a meta-analysis, AMR increased during the pandemic period, especially among Gram-negative bacteria in hospital settings [[22], [23], [24]].

Given the aforementioned evidence, we hypothesized that COVID-19 vaccination is associated with decreased antibiotic use in COVID-19 patients, thereby strengthening the argument for the important role of vaccination in combating AMR. Therefore, we aimed to explore the relationship between COVID-19 vaccination status and the use of antibiotics in COVID-19 patients. We conducted a systematic literature review to address this knowledge gap by synthesizing newly published evidence from 2021 to 2024. The research question of the current systematic review was:

• Is there a relationship between COVID-19 vaccination status and antibiotic use or prescriptions in COVID-19 patients? If so, what is the direction of this relationship?

## Methods

This systemic review is reported according to the latest PRISMA guidelines [25]. Τhe study protocol was submitted to PROSPERO (ID: CRD42023449625) registry before the initial screening of the literature.

### Eligibility criteria and search strategy

The eligibility criteria for the included articles were formulated using the Population, Intervention, Comparison, Outcomes, and Study (PICOS) framework [26]. Quantitative studies of all designs (S) addressing the relationship between COVID-19 vaccination (I) status (C) and antibiotic use (O) in COVID-19 patients (P), with or without secondary bacterial infections or co-infections, were eligible for inclusion. Only articles written in English were included. The search strategy was initially developed for the PubMed database and was subsequently translated for use in the Embase, Web of Science, Scopus and Google Scholar databases. The first 50 pages of Google Scholar were searched (10 results per page). The detailed search strings are presented in Supplementary Material, Table 1. Although no articles on the relationship between COVID-19 vaccination and antibiotic use were identified in the most recent relevant systematic review up to December 1, 2021, we decided to conduct a search covering an overlapping period, including additional databases such as Web of Science, Scopus, and Google Scholar [16]. We included articles published between January 1, 2021, and November 6, 2024, when the actual search was performed.

### Screening of evidence, data extraction and quality assessment of the included studies

EndNote software was used to manage the screening process and exclude duplicates. An independent, double-author screening (M.P. and G.R.) was employed for both title/abstract and full-text screening, as well as for data extraction. Any disagreements were resolved through consensus between the researchers. All included studies underwent an assessment for the risk of systematic bias using the Newcastle-Ottawa tool for cross-sectional, case-control and cohort studies. The evaluation was conducted independently by M.P. and G.R. Any discrepancies were resolved through consensus between the researchers. The Newcastle-Ottawa appraisal tool assesses seven or eight items across three domains: the selection of study groups, the comparability of the groups, and the ascertainment of either the exposure (for case-control studies) or the outcome (for cohort and cross-sectional studies) of interest. The studies may be awarded up to two stars for each item, with a maximum score of 10/10 for cross-sectional studies and 9/9 for case-control and cohort studies. As adopted by previous studies, we applied an overall cut-off of ≥7 for cross-sectional studies and >7 for cohort and case-control studies to classify the included studies as having high or low risk of systematic bias [27,28].

### Evidence synthesis

The main study characteristics and the results of the quality assessment were tabulated and presented by study design. A brief narrative synthesis was performed to summarize the results of the included studies. The DerSimonian-Laird random-effects meta-analysis model was used to estimate the pooled odds ratio (OR) and the 95% confidence interval (CI). We opted to use the random-effects model to account for heterogeneity, given the differences in study designs, participant characteristics, and geographical disparities in the included studies. ORs were either provided by the individual studies or calculated from the reported raw data. If an adjusted OR was provided by a study, it was preferred over the unadjusted OR for inclusion in the analysis. When data on each vaccination dose were available, patients were considered vaccinated if they had completed two doses of the COVID-19 vaccine. When needed, the standard errors were calculated according to the formula provided by Altman and Bland [29]. The between-study heterogeneity was quantified using the I^2^ statistic, and the Cochrane Q statistic was employed to assess heterogeneity between studies and subgroups. To further investigate the observed statistical heterogeneity, we conducted a subgroup analysis based on study design. To evaluate the robustness of our results, we performed three sensitivity analyses: (i) excluding studies with a higher risk of systematic bias, (ii) excluding the two studies conducted among solid organ transplant recipients, and (iii) pooling odds ratios corresponding to the highest number of COVID-19 vaccine doses reported by the individual studies. The small study effect was assessed both qualitatively and quantitatively with a funnel plot and Egger's test. Stata Statistical Software (College Station, TX: StataCorp LLC) version BE 18 was used for all statistical analyses.

## Results

**Figure 1:**
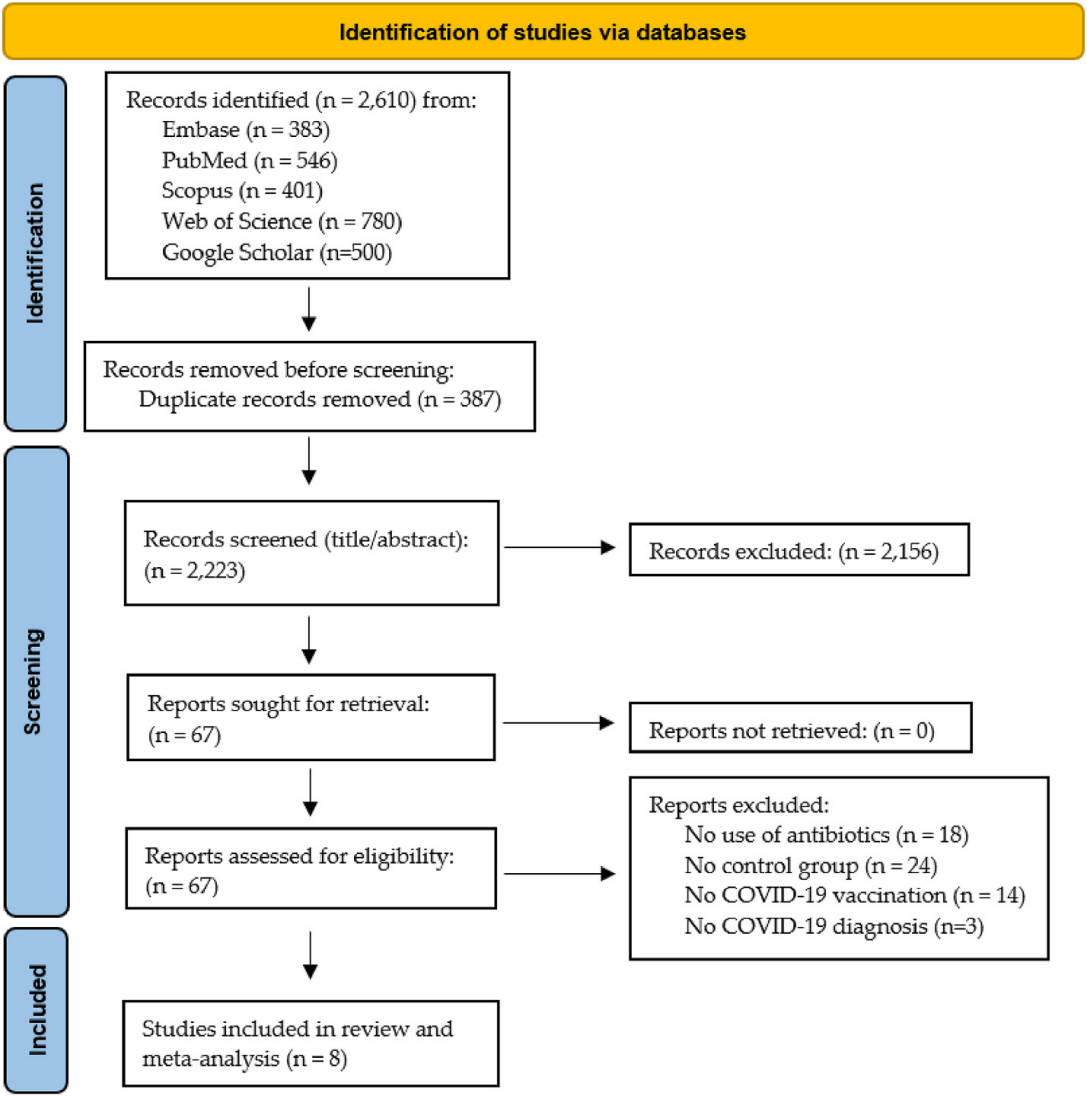
Flow-chart of the systematic review according to the PRISMA guidelines.

The PRISMA flowchart is depicted in Figure 1. A total of 2,610 articles were identified through the database searches. After excluding duplicates, 2,223 articles were screened during the title/abstract screening, resulting in the exclusion of 2,156 articles. During the full-text screening of the remaining 67 articles, 59 articles were excluded for specific reasons. A total of eight articles were deemed eligible for inclusion in the review and meta-analysis [17,[30], [31], [32], [33], [34], [35], [36]].

### Quality assessment of the included studies

All included studies were evaluated for the risk of systematic bias using the Newcastle-Ottawa appraisal tool (Table I). All cross-sectional studies were found to have a high risk of systematic bias, primarily due to the lack of confounding control and the use of non-representative participant samples. The sole case-control study was assessed as having a high risk of systematic bias, while all three cohort studies were appraised as being of very high quality.

**Table I:** Quality assessment of the included studies by study design according to the Newcastle-Ottawa appraisal tool

**Cross-sectional studies**	Selection (maximum 5 stars)				Comparability (maximum 2 stars)	Outcomes (maximum 3 stars)			Score (Max. 10)
	Representativeness of the sample	Sample size	Non-respondents	Ascertainment of the exposure (risk factor)		Assessment of the outcome	Statistical test		

Biros *et al.*		∗	∗	∗∗		∗∗			6/10
Chaaban *et al.*		∗							1/10
Dedic *et al.*	∗	∗	∗			∗∗			5/10
Despotovic *et al.*	∗	∗	∗			∗∗	∗		6/10

**Case-control studies**	Selection (maximum 4 stars)				Comparability (maximum 2 stars)	Exposure (maximum 3 stars)			Score (Max. 9)
	Αdequate case definition	Representativeness of the cases	Selection of Controls	Definition of Controls		Ascertainment of exposure	Same method of ascertainment for cases and controls	Non-Response rate	

Demir *et al.*	∗		∗	∗		∗	∗	∗	6/9

**Cohort studies**	Selection (maximum 4 stars)				Comparability (maximum 2 stars)	Outcomes (maximum 3 stars)			Score (Max. 9)
	Representativeness of the exposed cohort	Selection of the non-exposed cohort	Ascertainment of exposure	Outcome not present at start of study		Assessment of the outcome	Adequate follow-up period	Adequacy of follow up	

Blais *et al.*	∗	∗	∗		∗∗	∗	∗	∗	8/9
Hall *et al.*	∗	∗	∗	∗	∗∗	∗	∗		9/9
MacFadden *et al.*	∗	∗	∗		∗∗	∗	∗	∗	8/9

### Main findings

Eight studies of various quantitative study designs (cross-sectional, case-control, and cohort studies) with 134,022 participants were included in the current systematic review and meta-analysis. Studies from Europe, Asia, and North America were included, the majority of which focused on hospitalized COVID-19 patients. All studies were published between 2022 and 2024 (Table II).

**Table II:** Main characteristics and findings of the included studies by study design

Study	Country	Participants	Exposure definition (COVID-19 vaccination)	Outcome of interest
**Cross-sectional studies**				
Biros *et al.* (2024)	Greece	COVID-19 hospitalized patients from the Infectious Diseases Unit at the University Hospital of Ioannina, Greece from January 2021 to September 2022 (n= 1107). Age (mean years): 65.2 Male/female 56.6/43.4%	Not described	Being vaccinated against COVID-19 decreased the initiation of antibiotic treatment within 72 hours of hospitalization, OR: 0.56, p < 0.001 Secondary bacterial infections or co-infections: Not reported
Chaaban *et al.* (2024)	Lebanon	Enrolled students of the Islamic University of Lebanon, as well as people from their close environment, with a history of at least one COVID-19 infection (n=478) Mean age was 26.6 years [SD 9.44 years] Male/female 28.2/71.8%	Participants who received one or more doses of a COVID-19 vaccine.	Antibiotic use during COVID-19 infection: Vaccinated: 38% Unvaccinated: 48.9% *P*= 0.053 Secondary bacterial infections or co-infections: Not reported
Dedic *et al.* (2022)	Bosnia and Herzegovina	COVID-19 patients admitted to the primary health care COVID-19 centre of Canton Sarajevo (n=400)	Participants who received one or more doses of a COVID-19 vaccine.	Usage of antibiotics during the initial admission for COVID-19 patients: 79.2% in COVID-19 vaccinated 77.1% in COVID-19 unvaccinated Chi-squared test *P*-value at a significance level of 95%: 0.61 Secondary bacterial infections or co-infections: Not reported
Despotovic *et al.* (2022)	Serbia	Inpatients with COVID-19 who were hospitalised in the Clinic for Infectious and Tropical Diseases, University Clinical Centre of Serbia (n=523) Age (mean ± SD): 56.7 ± 16.0 Female: 40.9%	Not described	Pre-Admission Antibiotic Use was positively associated with negative COVID-19 vaccination status: Vaccinated: 50.7% Unvaccinated: 60.7% Chi-squared test *P*-value at a significance level of 95% = 0.04 Bacterial co-infections: 3% of the participants
**Case-control studies**				
Demir *et al.* (2022)	Turkey	Vaccinated kidney transplant recipients with COVID-19 from two transplant centres were matched using propensity score (Age, gender, type of donor, time from transplantation to COVID-19 diagnosis) with an unvaccinated kidney transplant recipient diagnosed with COVID-19 (n=164)	Participants who received at least two doses of either CoronaVac (Sinovac Life Sciences) or BNT162b2 (Pfizer-BioNTech)	Use of antibiotics: 22.5% in vaccinated patients 46.3% in the control group (unvaccinated) Chi-squared test *P*-value at a significance level of 95%: <0.001 Secondary bacterial infections or co-infections: Not reported
**Cohort studies**				
Blais *et al.* (2023)	Hong-Kong	All patients with community-acquired COVID-19 who were admitted to public hospitals in Hong Kong (n= 65,810). Median age in years (IQR): 70.0 (44.0,84.0).	Participants were divided into five categories: 0 doses, 1 dose, 2 doses, 3 doses, and 4 doses.	COVID-19 vaccine received 14 days prior to admission and inpatient antibacterial prescription: 1 COVID-19 vaccine dose: OR 1.01 (0.93–1.08) 2 COVID-19 vaccine doses: OR 0.74 (0.69–0.78) 3 COVID-19 vaccine doses: OR 0.69 (0.64–0.74) 4 COVID-19 vaccine doses: OR 0.52 (0.44–0.62) ORs adjusted for patient characteristics and disease severity Secondary bacterial infections or co-infections: Not reported
Hall *et al.* (2022)	Canada	Solid organ transplant recipients with PCR confirmed SARS-CoV-2 infection were eligible to be included in the analysis. Unvaccinated participants were matched with propensity score to vaccinated participants (prior SARS-CoV-2 infection, age, sex, transplant type, and number of comorbidities), n=297.	Participants who received one or 2 doses of a COVID-19 vaccine.	Prescription of any antibiotic: 24.7% in vaccinated patients 29.5% in unvaccinated patients Bivariate analysis, *P*-value=0.706 Bacterial co-infection: 13% in vaccinated, 5.0% in unvaccinated patients, p = 0.036.
MacFadden *et al.* (2023)	Canada	Individuals with a first laboratory-confirmed identification of SARS-CoV-2 among all residents aged ≥66 years, across the province of Ontario (nursing home residents=13,529, community residents=50,885).	Participants who received two or more doses of a COVID-19 vaccine.	Antibiotic prescription rates: Vaccinated individuals had significantly lower adjusted IRRs of antibiotic prescription in outpatient settings when compared to unvaccinated individuals in both pre-diagnosis (IRR: 0.43; 95%CI: 0.37–0.51) and postdiagnosis period (IRR: 0.31; 95%CI: 0.26–0.37). Vaccinated nursing home residents had a decreased (IRR: 0.66; 95%CI: 0.44–0.99) of antibiotic prescription during the post-diagnosis period while no difference in the IRR of antibiotic prescription was described between vaccinated and unvaccinated nursing home residents during the pre-diagnosis period (IRR 1.18; 95%CI: 0.78–1.78) IRRs adjusted for: demographics, comorbidities, health care utilization measures, provider characteristics, facility characteristics, residents' health and functional status, place of residence-level social determinants of health Secondary bacterial infections or co-infections: Not reported

Four studies of cross-sectional design were included in this systematic review. A study conducted in Bosnia and Herzegovina (Dedic *et al.*) involving 400 COVID-19 patients found no difference in antibiotic use during initial admission to a primary care health centre based on COVID-19 vaccination status. Specifically, 79.2% of vaccinated patients and 77.1% of unvaccinated patients received antibiotics (*P*-value = 0.61). A study from Serbia (Despotovic *et al.*) which included 523 participants showed that COVID-19 vaccination status was negatively associated with the use of antibiotics in COVID-19 patients before admission (60.7% among unvaccinated and 50.7% among vaccinated, *P*=0.04). In a study from Greece, Biros *et al.* reported that being vaccinated against COVID-19 decreased the initiation of antibiotic treatment in hospitalized COVID-19 patients (OR: 0.56; *P* < 0.001). Lastly, 38% of the vaccinated and 48.9% of the unvaccinated participants in a survey (Chaaban *et al.*), mostly enrolled students at the Islamic University of Lebanon, reported using antibiotics during a COVID-19 infection (*P* = 0.053).

In the only case-control study (Demir *et al.*) investigating all-cause mortality between vaccinated and unvaccinated kidney transplant recipients with COVID-19 (n=164) conducted in Turkey, being vaccinated with a COVID-19 vaccine was negatively associated with the use of antibiotics, based on the results of bivariate analysis (22.5% in the vaccinated group and 46.3% in the unvaccinated group; *P*-value<0.001). The two groups of participants were similar in terms of age, gender, type of donor and time from transplantation to COVID-19 diagnosis, as determined by propensity score matching.

Regarding cohort studies, a population-based study from Hong Kong (Blais *et al.*) with 65.810 participants found that each additional COVID-19 vaccine dose was associated with a significant decrease in the adjusted odds of inpatient antibiotic prescription by 26% (second dose), 31% (third dose), and 48% (fourth dose). In a study primarily investigating the severity and mortality outcomes of COVID-19 between vaccinated and unvaccinated solid organ transplant recipients in Canada (Hall *et al.*), antibiotic prescriptions did not differ based on the results of bivariate analysis (n=297; 24.7% in vaccinated patients, 29.5% in unvaccinated patients; *P*-value=0.706). In the latter study participants were matched according to their COVID-19 vaccination status with a propensity score matching (prior SARS-CoV-2 infection, age, sex, transplant type, and number of comorbidities). A population-based study from Canada (MacFadden *et al.*) with 64.414 participants aged 66 years and older found that being vaccinated against COVID-19 significantly lowered the risk of antibiotic prescription in outpatient clinics both in pre- and post- COVID-19 diagnosis period (adjusted IRR: 0.43; 95%CI: 0.37–0.51 and adjusted IRR: 0.31; 95%CI: 0.26–0.37, respectively), after adjustments for important confounders. According to the same study, being vaccinated against COVID-19 was associated with a lower risk of antibiotic prescriptions in nursing home residents during the post-diagnosis period (adjusted IRR: 0.66; 95%CI: 0.44–0.99) but no difference was identified during the pre-diagnosis period (adjusted IRR 1.18; 95%CI: 0.78–1.78).

### Meta-analysis: main analysis, subgroup analysis, and sensitivity analysis

**Figure 2:**
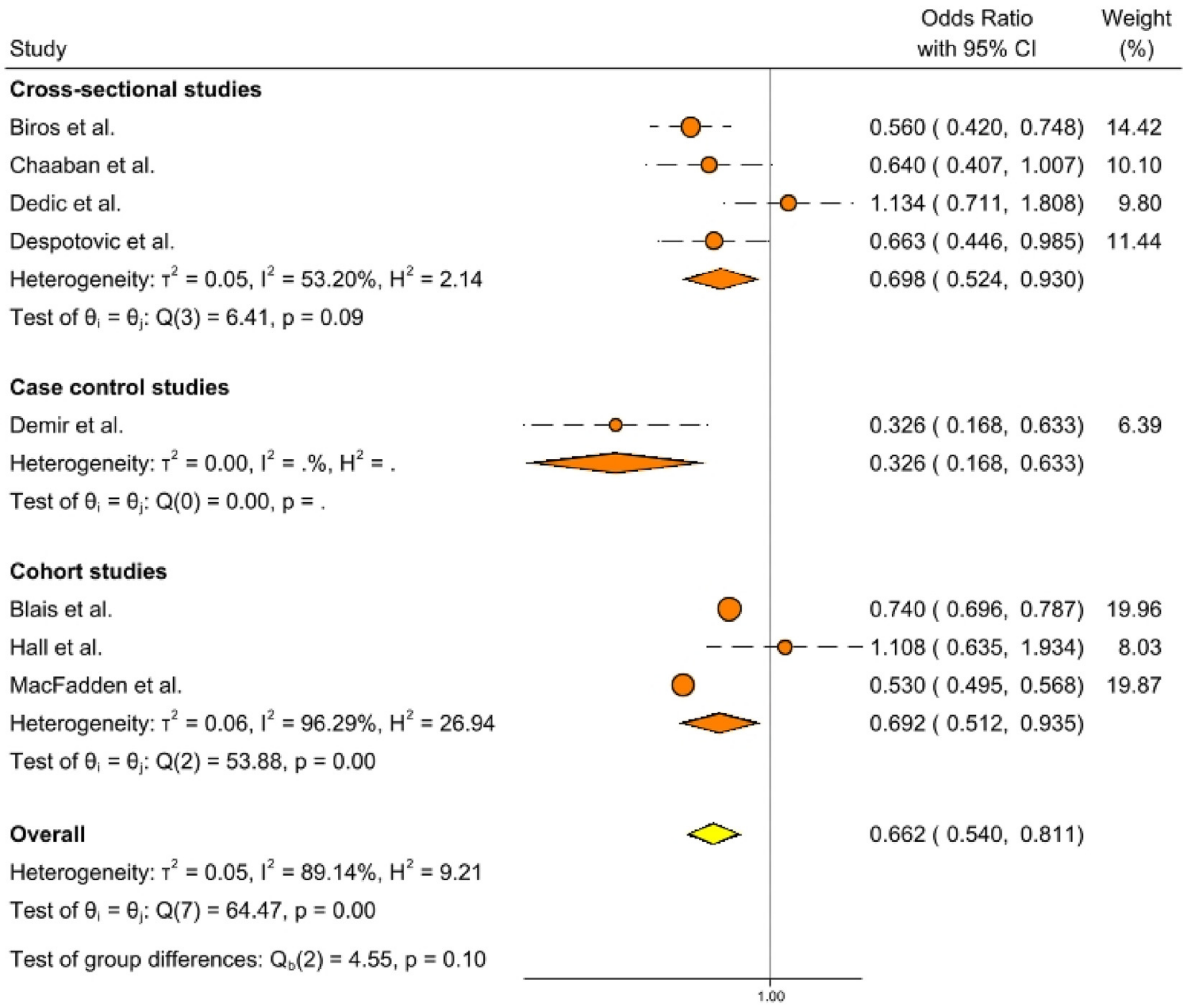
The results of the main analysis and subgroup analysis regarding the relationship between COVID-19 vaccination status and the odds of antibiotic use in COVID-19 patients. Orange circles represent individual studies, orange diamonds represent pooled group effect sizes with 95% CI, and the yellow diamond represent the overall pooled effect size with 95% CI.

Figure 2 displays the results of the main analysis. Vaccination with a COVID-19 vaccine was significantly associated with a 34% reduction in the odds of antibiotic use or prescriptions in COVID-19 patients (OR: 0.662; 95% CI: 0.540–0.811). The overall between-study heterogeneity was high, with an I^2^ statistic of 89.14% (*P*=0.00). In the subgroup analysis, a significant negative association was observed between COVID-19 vaccination and antibiotic use among COVID-19 patients across all study designs. Specifically, the decrease was 30% in cross-sectional studies (OR: 0.698; 95% CI: 0.524–0.930), 31% in cohort studies (OR: 0.692; 95% CI: 0.512–0.935), and 67% in the sole case-control study (OR: 0.326; 95% CI: 0.168–0.633). The I^2^ was moderate (53.20%; *P*=0.09) in cross-sectional studies and high (96.29%; *P*=0.00) in cohort studies.

In the sensitivity analysis, among studies with a lower risk of systematic bias, the pooled OR of antibiotic use was 0.692 (95% CI: 0.512–0.935), with high between-study heterogeneity (I^2^ = 96.29%; *P*=0.00) [17,31,36]. After excluding the two studies conducted among solid organ transplant recipients, the pooled OR was 0.664 (95% CI: 0.536–0.821), with high between-study heterogeneity (I^2^ = 91.19%; *P*=0.00) [17,[30], [31], [32], [33],^35^]. Finally, when pooling data for participants receiving the highest reported number of COVID-19 vaccine doses in the individual studies, the pooled OR was 0.609 (95% CI: 0.512–0.723), with substantial between-study heterogeneity (I^2^ = 65.63%; p = 0.00) [17,[30], [31], [32], [33], [34], [35], [36]].

### Publication bias assessment

**Figure 3:**
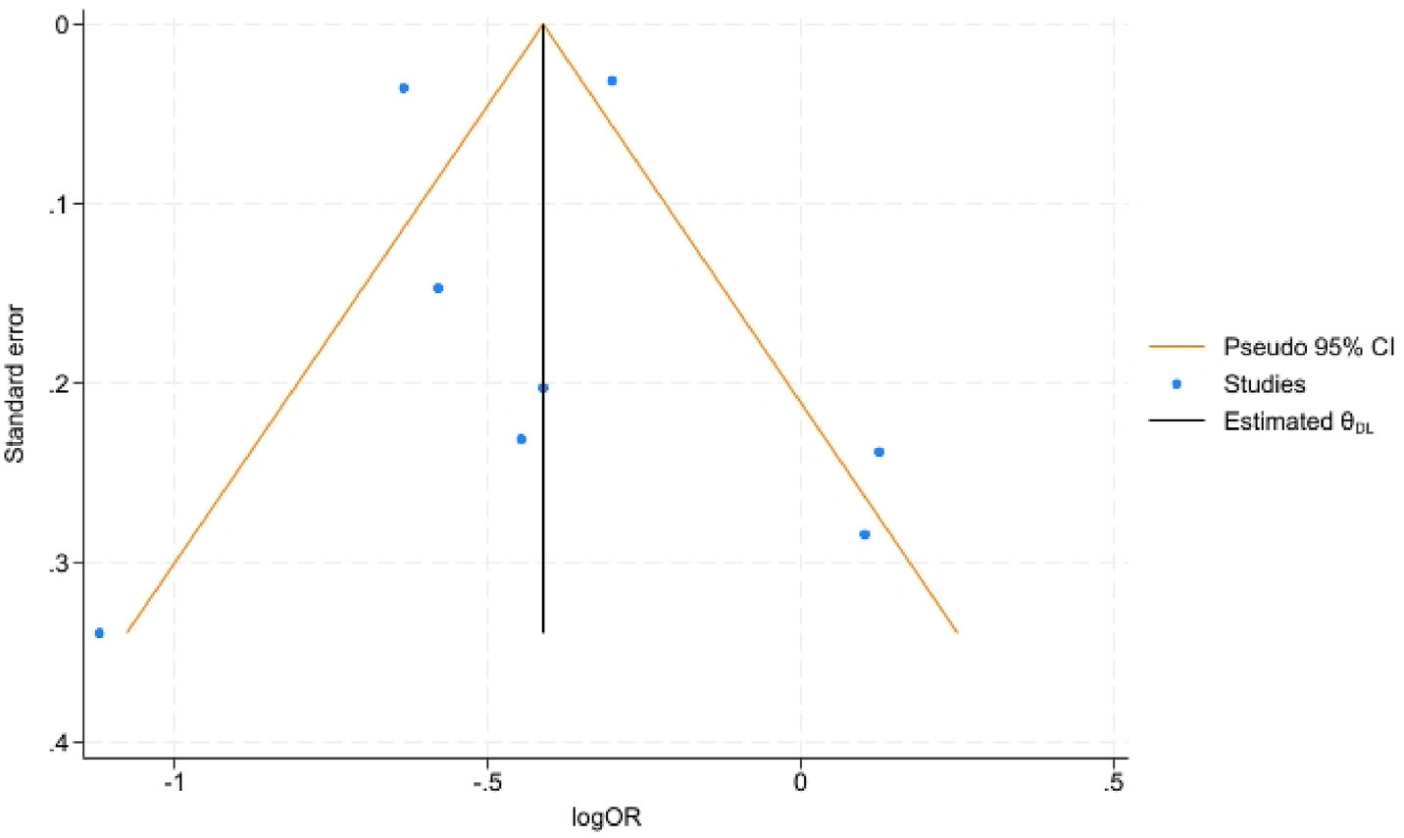
The funnel plot shows the effect size of each study (logOR) on the x-axis plotted against their standard error on the y-axis. The black vertical line represents the pooled effect size, while the orange lines indicate the pseudo 95% CI. The blue dots represent the individual studies.

No small study effect was identified in our analysis, as no asymmetry was observed in the funnel plot (Figure 3). In alignment, the Egger's test did not identify any significant small study effect (*P*-value = 0.74).

## Discussion

We conducted a systematic literature review between 2021 and 2024 to explore the association of COVID-19 vaccination status with antibiotic use in COVID-19 patients. We identified eight relevant articles based on pre-specified eligibility criteria. Being vaccinated against COVID-19 was found to be significantly associated with decreased odds of antibiotic use in COVID-19 patients (OR 0.662; 95% CI 0.540–0.811). In the subgroup analysis, a significant negative relationship between COVID-19 vaccination and the odds of antibiotic use was found across all study designs (cohort studies: OR 0.692; 95% CI 0.512–0.935; cross-sectional studies: OR 0.698; 95% CI 0.524–0.930; and the single case-control study: OR 0.326; 95% CI 0.168–0.633). In all sensitivity analyses, COVID-19 vaccination was significantly associated with reduced odds of antibiotic use. The risk of systematic bias was found to be high in cross-sectional studies and low in cohort studies. No small-study effect was detected in our analysis.

Our results align with the findings of previous meta-analyses that investigated the effect of influenza and pneumococcal vaccination on antibiotic prescriptions or use. As discussed, evidence from a meta-analysis of RCTs showed that influenza vaccination reduced antimicrobial prescriptions or days of antibiotic use with a RoM of 0.71 (95% CI: 0.62–0.83), while also decreasing the proportion of people receiving antibiotics with an RR of 0.63 (95% CI: 0.51–0.79) [16]. Furthermore, a significant effect of pneumococcal vaccination on lowering the number or days of antimicrobial prescriptions in children was found (RoM = 0.94; 95% CI: 0.92–0.96), while the same trend was also reported for both pneumococcal and influenza vaccines, based on the results of 69 observational studies [16]. The authors of the latter study mentioned that they did not conduct a meta-analysis of observational studies due to the variability observed in the study characteristics. However, the necessity of a complementary meta-analysis of observational studies for such an outcome should be noted, while the observed heterogeneity could be further investigated using meta-analytical methods (subgroup analysis, meta-regression). The participants' risk-benefit ratio in RCTs is primarily assessed on an individual level [[37], [38], [39]]. Reducing antibiotic use/prescriptions is not expected to yield a significant benefit for an RCT participant, as antibiotics demonstrate a good safety profile, with allergic reactions being the most common adverse effects [40]. This may explain why the effect of vaccination on antibiotic use/prescriptions was not the primary outcome in almost all the RCTs included in the above-mentioned meta-analysis, even for extensively studied vaccines known for their favourable safety profiles (influenza, pneumococcal) [16,41,42]. Secondary and tertiary outcomes of an RCT are outcomes for which the RCT was not initially designed, making them vulnerable to false-positive and/or false-negative results, especially in cases of multiple testing [[43], [44], [45]]. Well-conducted, population-based cohort studies using registry data might be a more suitable study design for this outcome of interest, providing a high level of evidence while being able to infer a causal relationship [46,47].

A plausible explanation for our findings is that COVID-19 vaccination may play a protective role against bacterial co-infections and/or secondary bacterial infections (hereafter referred to as bacterial infections) in COVID-19 patients, analogous to the influenza vaccine, which indirectly protects against bacterial infections in influenza patients [5]. Based on that hypothesis, it can then be speculated that antibiotics were more frequently prescribed to unvaccinated COVID-19 patients with suspected or confirmed bacterial coinfections. However, evidence regarding the relationship between COVID-19 vaccination and the risk of bacterial infections in COVID-19 patients remains limited. A retrospective cohort study reported that the odds of secondary infections (including viral, bacterial, and fungal infections) were higher among unvaccinated hospitalized patients with COVID-19 or post-COVID-19 conditions (aOR 1.59; 95% CI: 1.45–1.75) [48]. Moreover, a retrospective cross-sectional study among hospitalized COVID-19 patients with a high prevalence of bacterial co-infections showed that there was no relationship between COVID-19 vaccination and co-infections *P*=0.056 (including all types of co-infections) [49]. Another cross-sectional study also reported that the odds of bacterial infections did not differ (OR: 1.36; 95% CI: 0.58–3.17) between vaccinated and unvaccinated COVID-19 patients admitted to the Infectious Disease Unit at Kenyatta National Hospital in Kenya [50]. Hall *et al.*, in their cohort study among solid organ transplant recipients, reported that confirmed bacterial infections occurred more frequently in vaccinated (13.0%) compared to unvaccinated (5.0%) COVID-19 patients (p = 0.036) [36]. The latter study is the only study that reported data on bacterial infections among vaccinated and unvaccinated participants in our review. In the same study, antibiotics were prescribed in 24.7% of vaccinated patients and 29.5% of unvaccinated patients. These figures indicate a possible trend of antibiotic overprescription in unvaccinated COVID-19 patients, even in the absence of bacterial infection. Up to now, given the limited evidence, the explanation that vaccinated COVID-19 patients have a decreased risk of bacterial infections compared to unvaccinated ones can only partially account for our findings.

Global antibiotic use increased during the COVID-19 pandemic, despite the documented low prevalence of bacterial infections among COVID-19 patients [19,51]. As previously discussed, results from meta-analyses showed that antibiotics were used in COVID-19 patients at rates far higher than the reported global prevalence of bacterial infections in this group [19,20]. Similarly, the rates of antibiotic use in the studies included in our review ranged from 25% to 79%, significantly exceeding the reported global prevalence of bacterial infections in COVID-19 patients [19]. Baghdadi *et al.*, in their cohort study among hospitalized COVID-19 patients (n = 64,691), found that 70.8% of the participants without a bacterial infection diagnosis received an antibiotic treatment [52]. Given that results from two RCTs showed no clinical benefit of antibiotic treatment with doxycycline or azithromycin in COVID-19 patients, the above-mentioned findings clearly indicate the irrational use of antibiotics in this patient group [53,54]. It can be speculated that antibiotics were predominantly used in unvaccinated COVID-19 patients for curative purposes against COVID-19 itself, possibly as an attempt to compensate for the protective benefits they lacked from COVID-19 vaccination. The latter explanation could be supported by the documented protective effects of COVID-19 vaccines in reducing severe outcomes (i.e., hospitalization, ICU admission, and mortality) in COVID-19 patients, as well as limited evidence from two cross-sectional studies from Pakistan and the Kingdom of Saudi Arabia showing that a substantial proportion of healthcare workers (58.1% and 36.9%) considered antibiotics to be an effective treatment for COVID-19 [[55], [56], [57], [58]]. In contrast, in a survey conducted between January and March 2019 among healthcare workers from 30 EU/EEA (European Union/European Economic Area) countries, only 1.7% of the participants responded that antibiotics were effective against viruses or against cold and flu [59]. The pressure for effective solutions during unexpected health crises, especially when evidence is lacking—as in the case of the COVID-19 pandemic—tends to increase inappropriate prescribing [60].

### Strengths and limitations

This is the first systematic review and meta-analysis examining the association between COVID-19 vaccination status and antibiotic use in COVID-19 patients, addressing a pivotal knowledge gap. We included studies from five databases, using an overlapping search period with the most recent systematic review, to achieve a more comprehensive search [16]. We synthesized evidence from eight observational studies with 134,022 participants and reported that COVID-19 vaccination was significantly associated with reduced odds of antibiotic use in COVID-19 patients. All sensitivity analyses replicated the significant negative relationship between the exposure and the outcome, thereby strengthening our main conclusion. The most weighted study (Blais *et al.*) contributed to the meta-analysis with an OR adjusted for various sociodemographic and clinical factors, further enhancing the reliability of our main and especially subgroup analysis. Lastly, the risk of publication bias in our analysis is considered very low.

A major limitation of our study is that the effect sizes of the individual studies were mostly derived from unadjusted analyses, which increases the risk of confounding in our analysis. However, as noted above, the most weighted study (Blais *et al.*) contributes to our results with an adjusted OR. Moreover, although the second most weighted study (MacFadden *et al.*) contributes with an unadjusted OR in our analyses, it aligns with the adjusted findings reported in the original study, thereby minimizing the risk of confounding bias in our analyses. Although subgroup analysis accounted for a substantial share of the observed between-study heterogeneity, it remained moderate to high, which may hinder the generalisability of our results. In our opinion, the observed statistical heterogeneity arises from the lack of confounding control, differences in study designs, diverse populations, differences in exposure definitions, and varying settings across countries. Lastly, due to the lack of data on bacterial infections among participants in the individual studies, we were unable to draw stronger conclusions from our findings.

### Conclusion

Vaccination with a COVID-19 vaccine was significantly associated with a 34% reduction in the odds of antibiotic use in COVID-19 patients. Large population-based cohort studies are needed to replicate these findings. COVID-19 vaccination status may have influenced healthcare providers' decisions regarding antibiotic use in this group. To draw more solid conclusions, further research on the relationship between COVID-19 vaccination and bacterial infections in COVID-19 patients is essential. Lastly, future studies should thoroughly investigate the factors contributing to the irrational prescribing of antibiotics in COVID-19 patients, particularly in relation to COVID-19 vaccination.

## Credit author statement

Conceptualization: M.P. Methodology: M.P., G.R., V.M., I.C. Formal analysis and data curation: M.P. Software: M.P. Writing–original draft preparation: M.P. Writing–review and editing: G.R., V.M., I.C. Supervision: G.R., V.M. Visualization: M.P. Validation: M.P.

## Funding statement

This research did not receive any specific grant from funding agencies in the public, commercial, or non-profit sectors. The Article Processing Charges were covered by the Karolinska Institute.

## Conflict of interest statement

The authors have no conflicts of interest to declare.
