# Supplementary material for: COVID-19 vaccination and use of antibiotics in COVID-19 patients: a systematic review and meta-analysis
Source: Infect Prev Pract. 2025 Jun 3;7(3):100461. doi: 10.1016/j.infpip.2025.100461 (PMC12414285; doi:10.1016/j.infpip.2025.100461)
Supplement: Supplementary file 1 [file mmc1.docx]

| PubMed | (("COVID-19 Vaccines"[Mesh]) OR (("SARS-CoV-2"[Mesh] OR "COVID-19"[Mesh]) AND ("Vaccination"[Mesh] OR "Immunization"[Mesh] OR "Immunization Programs"[Mesh] OR "Vaccination Coverage"[Mesh])) OR (("COVID-19"[tw] OR "COVID"[tw] OR "SARS-CoV-2"[tw] OR "coronavirus"[tw]) AND ("vaccin*"[tw] OR "immuni*"[tw] OR "inoculat*"[tw]))) AND ((("Anti-Bacterial Agents"[Mesh] OR "Anti-Infective Agents"[Mesh]) AND ("Prescriptions"[Mesh])) OR (("antimicrobial*"[tw] OR "anti-microbial"[tw] OR "antibiotic*"[tw] OR "anti-biotic*"[tw] OR "antibacterial*"[tw] OR "anti-bacterial*"[tw] OR "antiinfective*"[tw] OR "anti-infective*"[tw]) AND ("prescr*"[tw] OR "usage"[tw] OR "use"[tw] OR "using"[tw] OR "utili*"[tw] OR "administ*"[tw]))) Filters: English, from 2021 – 2024 |
| --- | --- |
| Embase | ('sars-cov-2 vaccine'/exp OR 'sars-cov-2 vaccine' OR (('severe acute respiratory syndrome coronavirus 2'/exp OR 'severe acute respiratory syndrome coronavirus 2' OR 'coronavirus disease 2019'/exp OR 'coronavirus disease 2019') AND ('vaccination'/exp OR 'vaccination' OR 'immunization'/exp OR 'immunization' OR 'preventive health service'/exp OR 'preventive health service')) OR (('covid-19' OR 'covid' OR 'sars-cov-2' OR 'coronavirus') NEXT/2 ('vaccin*' OR 'immuni*' OR 'inoculat*'))) AND (('antiinfective agent'/exp OR 'antiinfective agent') AND ('prescription'/exp OR 'prescription') OR (('antimicrobial*' OR 'anti-microbial' OR 'antibiotic*' OR 'anti-biotic*' OR 'antibacterial*' OR 'anti-bacterial*' OR 'antiinfective*' OR 'anti-infective*') NEXT/2 ('prescr*' OR 'usage' OR 'use' OR 'using' OR 'utili*' OR 'administ*'))) AND ([article]/lim OR [article in press]/lim OR [preprint]/lim) AND [english]/lim AND [2021-2024]/py |
| Scopus | ( ( "COVID-19 Vaccin*" ) OR ( ( sars-cov-2 OR covid-19 OR covid OR sars-cov-2 OR coronavirus ) PRE/2 ( vaccin* OR immuni* OR inoculat* ) ) ) AND ( ( antimicrobial* OR anti-microbial OR antibiotic* OR anti-biotic* OR antibacterial* OR anti-bacterial* OR antiinfective* OR anti-infective* ) PRE/2 ( prescr* OR usage OR use OR using OR utili* OR administ* ) ) AND PUBYEAR > 2020 AND PUBYEAR < 2024 AND ( LIMIT-TO ( DOCTYPE , "ar" ) ) AND ( LIMIT-TO ( LANGUAGE , "English" ) ) |
| Web of Science | (((("COVID-19 Vaccin*") OR ((SARS-CoV-2 OR COVID-19 OR COVID OR SARS-CoV-2 OR coronavirus) AND (vaccin* OR immuni* OR inoculat*))) AND ((antimicrobial* OR anti-microbial OR antibiotic* OR anti-biotic* OR antibacterial* OR anti-bacterial* OR antiinfective* OR anti-infective*) AND (prescr* OR usage OR use OR using OR utili* OR administ*)))) (All Fields) and 2024 or 2023 or 2022 or 2021 or 2020 (Publication Years) and Article or Early Access (Document Types) and English (Languages) and 2020 or 2021 or 2022 or 2023 or 2024 (Publication Years) and Article or Early Access (Document Types) and English (Languages) |
| Google Scholar | ((COVID-19 vaccine*) AND (use OR prescr*) AND (antibiotic* OR antimicrobial*)) |

**Table I.** Detailed search strings by database
